# The Impact of Abortion Underreporting on Pregnancy Data and Related Research

**DOI:** 10.1007/s10995-021-03157-9

**Published:** 2021-04-30

**Authors:** Sheila Desai, Laura D. Lindberg, Isaac Maddow-Zimet, Kathryn Kost

**Affiliations:** Guttmacher Institute, 125 Maiden Lane, 7th Floor, New York, NY 10038 USA

**Keywords:** Abortion, Pregnancy, Measurement, Data quality, NSFG

## Abstract

**Introduction:**

The impact on research findings that use pregnancy data from surveys with underreported abortions is not well-established. We estimate the percent of all pregnancies missing from women’s self-reported pregnancy histories because of abortion underreporting.

**Methods:**

We obtained abortion and fetal loss data from the 2006–2015 National Survey of Family Growth (NSFG), annual counts of births from US vital statistics, and external abortion counts from the Guttmacher Institute. We estimated the completeness of abortion reporting in the NSFG as compared to the external counts, the proportion of pregnancies resolving in abortion, and the proportion of pregnancies missing in the NSFG due to missing abortions. Each measure was examined overall and by age, race/ethnicity, union status, and survey period.

**Results:**

Fewer than half of abortions (40%, 95% CI 36–44) that occurred in the five calendar years preceding respondents’ interviews were reported in the NSFG. In 2006–2015, 18% of pregnancies resolved in abortion, with significant variation across demographic groups. Nearly 11% of pregnancies (95% CI 10–11) were missing from the 2006–2015 NSFG due to abortion underreporting. The extent of missing pregnancies varied across demographic groups and was highest among Black women and unmarried women (18% each); differences reflect both the patterns of abortion underreporting and the share of pregnancies ending in abortion.

**Discussion:**

Incomplete reporting of pregnancy remains a fundamental shortcoming to the study of US fertility-related experiences. Efforts to improve abortion reporting are needed to strengthen the quality of pregnancy data to support maternal, child, and reproductive health research.

## Significance

*What is known about this topic?* Despite being legal, abortion is a sensitive and stigmatized experience in the United States. It has been well-established that women do not fully report their abortions in individual-level surveys, compromising the study of abortion and pregnancy in the United States.

*What does this study add?* This study examines the share of missing pregnancies due to abortion underreporting in the National Survey of Family Growth (NSFG) and demonstrates how incomplete abortion reporting can impact research on pregnancy experiences, not just abortion. Our findings underscore the need to improve abortion reporting to strengthen the quality of overall pregnancy data to support maternal, child, and reproductive health research.

## Introduction

Abortion is a sensitive and stigmatized experience, even in the United States, where it is legal (Hanschmidt et al., 2016; Shellenberg & Tsui, 2012). It has been well-established that women^1^ do not fully report prior abortions in individual-level surveys. Much of this underreporting is likely driven by stigma, as respondents may deliberately omit stigmatized behaviors to provide more socially desirable responses (Tourangeau & Yan, 2007). Multiple studies document abortion underreporting in the NSFG, the premier survey of fertility behaviors in the US, and in other US surveys (Fu et al., 1998; Jones & Forrest, 1992; Jones & Kost, 2007; Lindberg et al., 2020a; Tierney, 2019). In response to this substantial underreporting, the NSFG User’s Guide has repeatedly provided cautionary guidance for over 2 decades advising against substantive research with the abortion survey items [National Center for Health Statistics (NCHS), 1997, 2020].

While it is evident that pervasive abortion underreporting impacts the study of abortion, little attention has been given to its impact on any study that relies on pregnancy data. Indeed, underreported abortions indicate underreported pregnancies, which can create biases in research that uses these reports. Furthermore, the extent of missing pregnancies likely varies across population groups, further biasing analyses. Previous analysis of the 2006–2015 NSFG estimated that 60% of abortions in the prior five calendar years were not reported in the face-to-face (FTF) interview compared with counts collected from abortion providers directly, with substantial variation across population groups (Lindberg et al., 2020a). However, this estimate of underreported abortions is not an adequate indicator of the magnitude of missing pregnancies in these data; the proportion of all pregnancies that are missing depends not only on the extent of abortion underreporting, but also on the share of pregnancies ending in abortion. Further, this share can vary for population groups. For example, for a given group, a high level of abortion underreporting could still result in a low proportion of missing pregnancies overall if few pregnancies end in abortion. Conversely, even for a population group with more complete abortion reporting, if the share of pregnancies ending in abortion for that group is large, the share of pregnancies missing from the data could be large.

In this paper, we examine nearly a decade’s worth of NSFG data and estimate the percent of all pregnancies missing from women’s self-reported pregnancy histories as a result of abortion underreporting, including by key demographic groups. We demonstrate that the implications of abortion underreporting extend beyond abortion research alone and caution researchers who may use these data in their analyses without addressing the consequences of incomplete abortion reporting.

## Methods

### Data Sources

We pooled data from the 2006–2010 and 2011–2015 NSFG, a nationally representative household survey of women and men aged 15–44 in the United States (Groves et al. 2009). The NSFG uses a multistage, stratified clustered sampling frame and all analyses presented here were weighted to account for the complex sampling design. Methods of data collection were reviewed by the NCHS Institutional Review Board protections for human subjects. Because this analysis used publicly available, de-identified data, the authors' Institutional Review Board granted this study exempt status.

We used abortion and fetal loss data from the FTF interviews, which collected a lifetime pregnancy history, including the outcome (live birth, abortion, or fetal loss) and date when the pregnancy ended. Fetal loss outcomes include stillbirth, miscarriage, and ectopic pregnancy. Miscarriages, in particular, are likely underreported in the NSFG (Lindberg & Scott, 2018) and are limited to recognized pregnancies (some fraction of pregnancies lost early in gestation are not recognized, and thus cannot be measured in self-reported data). We use NSFG reports of fetal loss, however, given the lack of data sources that accurately capture the incidence of miscarriage in the population.

Although the NSFG also collects information about pregnancies in the last five years in an audio computer assisted self-interview (ACASI), we do not use this measure given identified limitations of these ACASI responses (Lindberg & Scott, 2018).^2^ Moreover, the FTF measures are more often used by researchers than the ACASI measures, as the latter do not include specific pregnancy dates or other details collected in the full pregnancy history.

We obtained the annual incidence of births from US vital statistics [U.S. Department of Health and Human Services (DHHS), 2018]. The incidence of abortion each year was obtained from the Guttmacher Institute’s Abortion Provider Census, which collects data directly from abortion providers and is designed to measure both surgical and medication abortions (Jones & Jerman, 2014, 2017). These are considered the most complete abortion counts available, as not all states report data to the Centers for Disease Control and Prevention (Jatlaoui, 2018). A small number of abortions are missed by the census, either because women obtain abortions from unidentified private practice physicians, or unidentified hospital settings (Jones & Kost, 2018), or because of the use of self-managed abortion (Ralph et al., 2020).

Both sets of counts were adjusted to match the NSFG’s sampling frame and five year recall period, using an approach described elsewhere (Lindberg et al., 2020b).

### Analytic Strategy

We first calculated the number of pregnancies occurring in the US by combining the corresponding external birth and abortion counts, and fetal losses from the NSFG. This approach of combining these data sources is informed by the pregnancy surveillance methodology^3^ used in the past by the NCHS (Ventura et al., 2012). As noted above, both the proportion of abortions missing from the data and the proportion of pregnancies ending in abortion impact the proportion of pregnancies missing in the data. For the proportion of abortions missing, overall and for population groups, we utilize previously published estimates (Lindberg et al., 2020a). We assessed the proportion of pregnancies ending in abortion, overall and for population groups, by calculating the ratio of the external abortion counts to the total pregnancy counts.

To estimate the percent of pregnancies missing in the NSFG, we calculated the ratio of missing abortions to all pregnancies. Missing abortions are calculated as the difference between the external and NSFG-reported counts of abortions. Because missing abortions mean missing pregnancies, this ratio reflects the share of all pregnancies that are missing as a result of unreported abortions. We calculated 95% confidence intervals and assessed significant differences between demographic groups on the basis of non-overlapping intervals. (Note that this approach is relatively conservative, as it will fail to reject the null hypothesis that the point estimates are equal more frequently than formal significance testing).

All counts and measures were calculated separately for population groups defined by age, race combined with Hispanic ethnicity, and union status; these are the only demographic variables that could be matched across vital birth records data, external abortion data, and the NSFG. Additionally, we compared estimates in the 2006–2010 and 2011–2015 survey rounds.

## Results

### Abortion Underreporting and Pregnancy Resolution Patterns

Fewer than half of abortions (40%, 95% CI 36–44) in the five calendar years preceding respondents’ interviews were reported in the 2006–2015 NSFG compared to external counts (Table 1). Estimates for age groups had non-overlapping confidence intervals, with more complete reporting among women younger than age 20. In contrast, differences in reporting for groups varying by race/ethnicity or union status or education were not statistically significant. The completeness of abortion reporting did not differ between the 2006–2010 and 2011–2015 survey periods.

**Table 1:** Percent of abortions reported in five calendar years prior to year of interview relative to adjusted external counts, percent of pregnancies ending in abortion, and percent of pregnancies missing from the data; by respondents’ characteristics and survey years, NSFG 2006–2015

	2006–15 NSFG				
	% Abortions reported^a^ (NSFG count of abortions/external count of abortions)	95% CI	% Pregnancies resolving in abortion (external count of abortions/pregnancies)	% Pregnancies missing (missing abortions/pregnancies)	95% CI
Total	40	36–44	18	11	10–11
Age at abortion					
< 20	53	43–62	26	12	10–15
20–29	37	32–42	20	13	12–14
30 and older	40	31–48	12	7	6–8
Race/ethnicity					
White, non-Hispanic	42	35–50	13	7	6–8
Black, non-Hispanic	41	33–48	29	18	15–20
Other, non-Hispanic	29	16–41	21	15	12–18
Hispanic	40	32–48	19	11	10–13
Union status					
Unmarried	41	37–46	31	18	17–20
Married	34	24–45	5	3	3–4
Survey year					
2006–2010	39	33–45	19	11	10–12
2011–2015	41	35–47	17	10	9–11

^a^Estimates from Lindberg et al., 2020a. Union status estimates have been adapted to reflect categories used in this analysis

Overall, external surveillance counts indicate that 18% of pregnancies in 2006–2015 resolved in abortion, with significant variation across demographic groups. A larger share of pregnancies ended in abortion among younger women, especially adolescents, among whom over one-quarter of all pregnancies resolved in abortion. Similarly, nearly one-third of all pregnancies among unmarried women ended in abortion compared to 5% of pregnancies among married women. The proportion of pregnancies ending in abortion varied by race/ethnicity, with non-Hispanic white women the least likely to have a pregnancy end with abortion. We found no variation between survey periods.

### Proportion of Pregnancies Missing from the NSFG Data

Overall 11% of pregnancies (95% CI 10–11) were missing from the 2006–2015 NSFG as a result of abortion underreporting and the proportion of pregnancies resolved by abortion. And, we found significant differences across demographic groups. For example, older women had a lower proportion of missing pregnancies than younger women; 7% (95% CI 6–8) of pregnancies were missing among women ages 30–44 compared to 12% (95% CI 10–15) among women ages 15–19 and 13% (95% CI 12–14) among those ages 20–29.

A smaller share of pregnancies were missing among non-Hispanic white women compared to other women [7% (95% CI 6–8) vs. 18% of non-Hispanic Black women (95% CI 15–20), 15% among women of other races (95% CI 12–18), and 11% among Hispanic women (95% CI 10–13)]. Although the proportion of abortions reported across these four race and Hispanic ethnicity groups varies, differences in the share of missing pregnancies more closely align with the patterns in the proportion of pregnancies resolving in abortion. Additionally, the percentage of missing pregnancies among unmarried women was 18% (95% CI 17–20) compared to approximately 4% (95% CI 3–4) among married women, reflecting both the higher ratio of abortions to pregnancies for unmarried women and their less complete abortion reporting. Finally, the proportion of pregnancies missing was comparable between the two survey periods.

## Discussion

We estimate that more than one in ten pregnancies are missing from the 2006–2015 NSFG data due to missing abortions. We also find that the share of missing pregnancies varies widely across demographic groups—a result of differential patterns in completeness of abortion reporting and the frequency of abortion relative to other pregnancy outcomes. A greater share of pregnancies are missing from the survey data among younger women, unmarried women, and women of color—demographic groups that are often the focus of pregnancy-related research and health policies. Without better quality pregnancy data, efforts to study and improve pregnancy-related health outcomes, including maternal and child health, are at risk of relying on biased and potentially misleading findings.

This study has limitations. First, without reliable external population-based data on the incidence of fetal loss, we include reported fetal losses from the NSFG in our calculation of pregnancies. This approach aligns with prior research, which found that NSFG miscarriage counts, for example, were comparable to those documented in prospective studies of pregnancy outcomes (Jones & Kost, 2007). Still, fetal losses, and miscarriages in particular, possibly are underreported to some extent in the NSFG (Lindberg & Scott, 2018), suggesting that our estimates may be conservative and provide a lower bound of the extent of missing pregnancies in NSFG data. The census abortion counts also may modestly undercount the true incidence of abortion in the US (Jones & Kost, 2018); this means that the ratio of abortions reported in the NSFG to the census counts may actually be smaller than our calculations, and the true number of missing pregnancies is actually larger than the estimates we calculated here. Further, confidence intervals for our estimates of the proportion of pregnancies that are missing from the data may be too narrow given they do not account for sampling error in estimated fetal loss counts from the NSFG. As a result, we are unable to make strong conclusions about differences between groups. However, this limitation does not impact the central focus of our analysis, which is to illustrate how abortion underreporting and patterns of pregnancy resolution jointly influence the extent of missing pregnancies.

Despite these limitations, the reach of this study’s findings is broad: abortion underreporting will impact not only studies that examine abortion, but also those that examine pregnancy. Moreover, data that provide an incomplete estimate of pregnancy experiences can bias analyses in a number of ways. First, they compromise research that utilizes pregnancy as an outcome. This issue is relevant both for population-level research, in which pregnancy counts will be too low, and individual-level studies, in which women’s experiences will be omitted from the analysis. Second, analytic models including abortion or pregnancy as a covariate also risk bias because of unmeasured confounding, as the unobserved factors associated with the likelihood of reporting may be correlated with other variables in the model. Third, these issues are further complicated because of the differential patterns of incomplete reporting, which makes biases unequal across groups. In addition, although we observed differential reporting for some key population groups, other differential reporting is also likely, including for characteristics that could not be measured in this study. As a result, these findings underscore the potentially significant biases of the NSFG pregnancy data and the caution necessary when using them.

Incomplete reporting of abortions also means that pregnancies ending earlier in gestation are omitted from pregnancy data, which means that pregnancy outcomes occurring later in pregnancy are disproportionately represented. This “survival bias” in pregnancy data can affect analyses used to inform and evaluate public health interventions or disparities (Ahrens & Hutcheon, 2020). For example, harmful environmental impacts on population health such as contaminated drinking water may be underestimated or undetected if pregnancy counts are incomplete and exclude miscarriages or abortions. As a result, the true effects of such environmental hazards on pregnancies may be, at best, not fully understood and, at worst, misleading, compromising efforts to protect and support maternal, infant, and child health (Nobles & Hamoudi, 2019). Additional research is needed to examine the impact of missing pregnancies on specific public health programming and policy-making.

This study examines the quality of pregnancy outcome data only in the NSFG, but this survey is not uniquely flawed in its level of underreporting of abortions and pregnancies. The detailed pregnancy histories collected in the NSFG enable us to examine these data and estimate their completeness more closely. Both the National Longitudinal Survey of Youth (NLSY) and the National Longitudinal Study of Adolescent to Adult Health have also been shown to have substantial abortion underreporting (Lindberg et al., 2020a). Indeed, because of widespread abortion stigma in the US (Bommaraju et al., 2016; Norris et al., 2011; Shellenberg & Tsui, 2012), we would expect incomplete reporting of abortion to be universal in this setting—whether in a large survey system (e.g., the NLSY) or a smaller clinical survey (Udry et al., 1996). Moreover, surveys outside of the US can also face these challenges of incomplete reporting in settings where abortion is legal but still socially sensitive (Saraç & Koç, 2020; Scott et al., 2019) or where it is illegal (Sedgh & Keogh, 2019). Researchers need to consider the completeness of abortion reporting and its potential impact on analyses when using data on pregnancy outcomes from any survey in which respondents are asked to report on all pregnancy outcomes.

Incomplete pregnancy reporting remains a fundamental shortcoming to the study of fertility-related experiences in the United States. Efforts to improve abortion reporting are needed to strengthen the quality of pregnancy data to support maternal, child, and reproductive health research. Complete data on pregnancies, not just births, enable accurate analyses on the wide range of pregnancy-related behaviors, experiences, and outcomes, and can inform the robustness of initiatives and interventions that seek to support the reproductive health of all.

## Data Availability

The data are publicly available.

## Footnotes

1 Only survey respondents self-identifying as female are asked about their pregnancy history. However transgender men, gender-nonbinary, and gender-nonconforming people also become pregnant and need and have abortions. We use the term “women” to describe survey respondents while acknowledging that these data limitations may exclude some people’s experiences.

2 For example, Lindberg & Scott, 2018 find that although the NSFG ACASI abortion measure was designed to measure the number of abortions in the 5 years prior to the interview, some women instead appear to report their lifetime number of abortions.

3 In brief, NCHS has previously calculated the total number of pregnancies in the US by combining live birth counts from birth certificate data, induced abortion counts from abortion surveillance data collected by CDC and the Guttmacher Institute and adjusted fetal loss estimates derived from the NSFG’s pregnancy history data.

## Code Availability

Available from the author on request.
